# Mobile-based in-home telerehabilitation compared with in-hospital face-to-face rehabilitation for elderly patients after total hip arthroplasty in China's level 1 trauma center: a noninferiority randomized controlled trial

**DOI:** 10.3389/fsurg.2024.1536579

**Published:** 2025-01-14

**Authors:** Yang Zhou, Yiming Lyu, Qiaojie Wang, Yanhong Ma, Lihua Huang, Xin Zhang

**Affiliations:** ^1^ Department of Orthopaedic and Sports Rehabilitation, The First Rehabilitation Hospital in Shanghai, Shanghai, China; ^2^ Department of Orthopedics, Medmotion Clinic, Shanghai, China; ^3^ Department of Orthopedics, Shanghai Sixth People’s Hospital Affiliated to Shanghai Jiao Tong University School of Medicine, Shanghai, China; ^4^ Department of Rehabilitation, Shanghai Sixth People’s Hospital Affiliated to Shanghai Jiao Tong University School of Medicine, Shanghai, China; ^5^ Rehabilitation Center, The First Rehabilitation Hospital in Shanghai, Shanghai, China; ^6^ School of Medicine, Tongji University, Shanghai, China

**Keywords:** THA, internet-based intervention, older adults, telerehabilitation, hip fracture

## Abstract

**Background:**

Telerehabilitation is gaining popularity in European and American countries, but whether it can be successfully implemented in China still lacks support from clinical studies.

**Objective:**

This trial aimed to determine if a home-based telerehabilitation method is clinically noninferior to standard in-hospital face-to-face rehabilitation for elderly patients with total hip arthroplasty (THA) in China.

**Methods:**

This multicenter randomized controlled trial was conducted from January 2021 to June 2022 at The First Rehabilitation Hospital in Shanghai, Shanghai Jiao Tong University affiliated Sixth People's Hospital and Shanghai Tongji University affiliated Tenth People's Hospital. Sixty-four patients were recruited for this two-arm, single-assessor blinded, randomized controlled trial. The participants were randomly assigned to the in-home telerehabilitation group (TELE group) and the in-hospital physical therapist in-person group (PT group). The intervention consisted of a 12-week home-based rehabilitation program with video instructions and remote coaching on a mobile APP (TELE group). The PT group received a standard in-hospital rehabilitation intervention assisted by a physical therapist for one month and outpatient clinic for the next two months. Patients were evaluated at baseline, 4 weeks, and 12 weeks postoperatively employing functional tests (Timed Up & Go test and Berg balance test) and self-reported questionnaires (Hip disability and Osteoarthritis Outcome Score (HOOS) and Short Form 12 (SF-12)).

**Results:**

There was no significant difference between the two groups for the demographic and clinical characteristics. 61 participants were analyzed (PT group: *n* = 31, women: 48.4% of participants; TELE group: *n* = 30, 33.3% of participants) whose median age was 70 and 69 years, in PT group (IQR: 63–73) and TELE group (IQR: 66–72) respectively. At 12 weeks follow-up evaluation, the main differences between the two groups regarding the HOOS gains, adjusted for baseline values, were close to zero (*P* > 0.05). There was no significant difference in primary and secondary outcome measures between the two groups.

**Conclusion:**

Our results showed the noninferiority of in-home telerehabilitation and advocated its application as a reliable alternative to in-hospital face-to-face rehabilitation for patients who underwent THA.

**Clinical Trial Registration:**

https://www.chictr.org.cn/, Chinese Clinical Trial Registry (Number: ChiCTR1900025825).

## Introduction

Physical rehabilitation following total hip arthroplasty (THA) is of particular importance, especially for elderly patients, as it can not only promote functional results but also reduce time spent on recovery (1). Due in part to the trend in the aging of the populations in big cities, which result in a high prevalence of hip fractures, the number of total hip replacement surgeries has steadily increased over the past decades while hospital stays have decreased (2). This patient group has consequently become a growing workload for physical therapists (PT) either in the community or hospital. In some communities in the author's home city (Shanghai, China), these patients group approximately accounts for over 30% of PT's caseload (3), and this number is increasing year by year. Since the growing rehabilitation needs of the elderly after THA cannot be met by the current labor force of PT in China, the exploration for new effective alternatives to ensure reliable and accessible postoperative physical rehabilitation is vital and urgent (4).

Another treatment model is to use telerehabilitation technology to deliver rehabilitation programs directly to the patient's home. This could help address access issues for patients living in rural and remote areas as well as those living in urban areas with transportation difficulties (5, 6). Many THA patients find it hard to access health care after they were discharged from the hospital. The elderly population coupled with postoperative implant dislocation, can make driving and transportation difficult. Access to rehabilitation programs is complex with patients' financial costs and health sectors providing in-home services in conjunction with or substitute with community care. For patients living in rural areas, the problem of access is compounded by long distances and time spent by patients or treating doctors. Technology-mediated home exercise programs may also encourage patients to exercise more frequently, potentially addressing the strength deficits that have been documented in patients with THA after surgery. In addition to addressing the problem of access, savings in the cost of providing health services are possible.

There have been some reports investigating telerehabilitation in postoperative rehabilitation programs of total knee arthroplasty (TKA) showed encouraging results (7). Besides achieving non-inferior outcomes when compared to conventional in-person rehabilitation programs, the majority of patients in telerehabilitation groups were satisfied with this alternative (5).

This study aims to determine whether telerehabilitation has similar effects as the conventional in-person rehabilitation program for patients who underwent THA.

## Methods

### Study design

A two-trauma-centers, randomized, single-blinded, two-armed, non-inferiority clinical trial compared telerehabilitation-based THA in-home rehabilitation program (TELE group) to traditional in-hospital PT care (PT group). The participants were recruited from January 2021 to June 2022 in two trauma centers (Shanghai Jiao Tong University affiliated Sixth People's Hospital and Shanghai Tongji University affiliated Tenth People's Hospital) and subsequently referred to The First Rehabilitation Hospital in Shanghai for rehabilitation treatment. Participants received a 12-week intervention and follow-up. Participants were evaluated at baseline (E1: at hospital discharge, one week post-operatively), after intervention (E2: four weeks after discharge), and three months later (E3: twelve weeks after discharge) by independent evaluators blinded to the staff of enrollment.

The study was registered in the Chinese Clinical Trial Registry (ChiCTR1900025825). All procedures were performed according to the Declaration of Helsinki, and the trial was approved by the Institutional Review Board (IRB) of The First Rehabilitation Hospital in Shanghai (IRB number: SHYK20180426-008). Other participating institutions and hospitals acknowledged the IRB approval. All participants signed a statement of informed consent after receiving clarifications regarding the study objectives and procedures. This study has been reported in line with Consolidated Standards of Reporting Trials (CONSORT) Guidelines (8).

### Participants

A total of 64 eligible patients were recruited for this two-arm single-assessor blinded randomized controlled trial. Inclusion criteria were as follows: (1) Patients admitted to one of the two trauma centers for a primary total hip arthroplasty with a diagnosis of an acute femoral neck fracture. (2) Patients are ≥60 years old. (3) Patients who can access Internet services after hospital discharge. (4) Patients who live within one hour of driving from either of the two trauma centers. Exclusion criteria were as follows: (1) Patients who have other combined injuries that could interfere with evaluation or the rehabilitation program. (2) Patients who had cognitive or collaboration problems. (3) Patients who had major postoperative complications. (4) Patients with severe cardiopulmonary conditions cannot tolerate rehabilitation programs.

### Intervention

A standard posterior surgical THA approach was used. The standardized rehabilitation intervention was based on the recommendations of a group of experts (9, 10), including 3 sessions per week of forty-five minutes to one hour; the intensity and duration were an assessment of the supervising PT according to each patient's tolerance and needs. Advice concerning pain control, walking aids, and the return to activities was also given to the patients.

**Figure 1:**
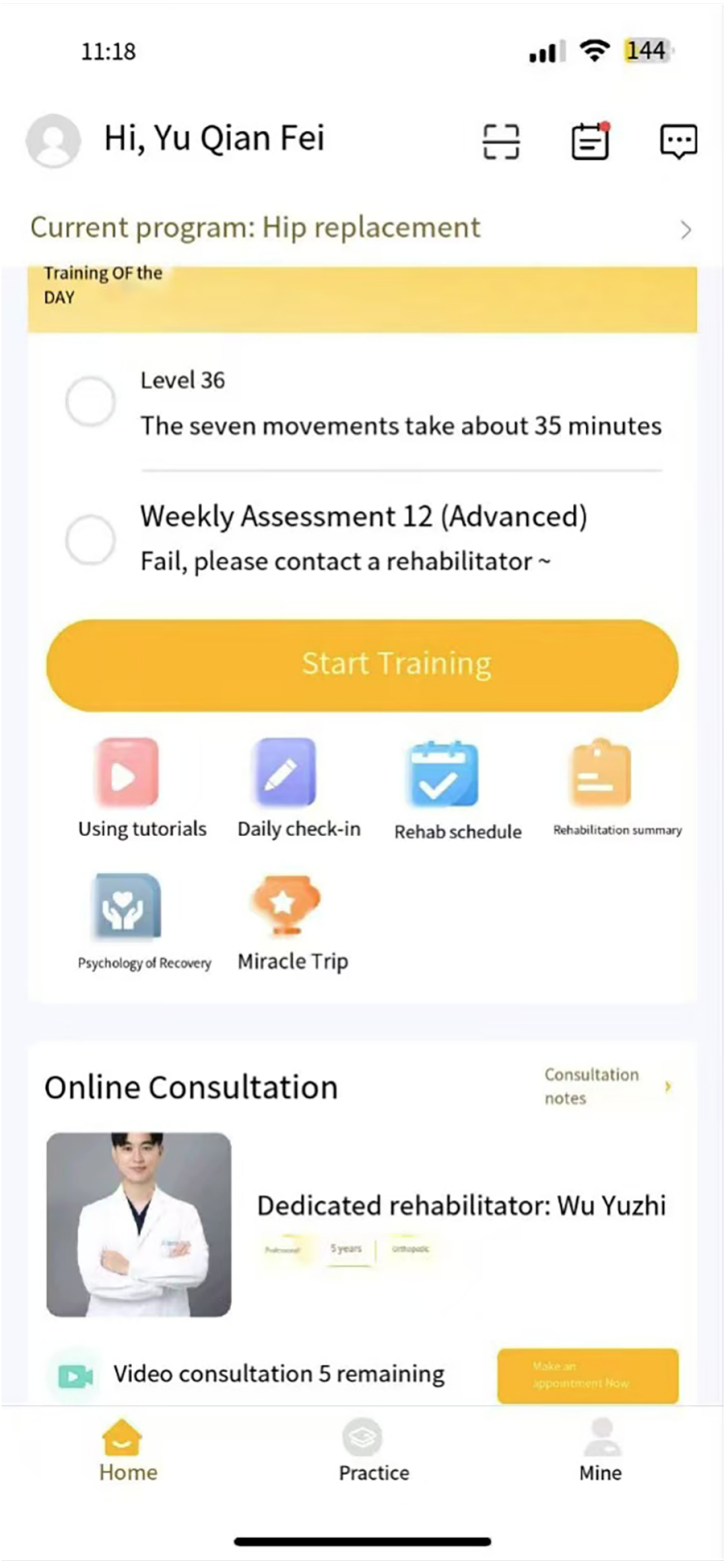
The user interface on the mobile APP.

**Figure 2:**
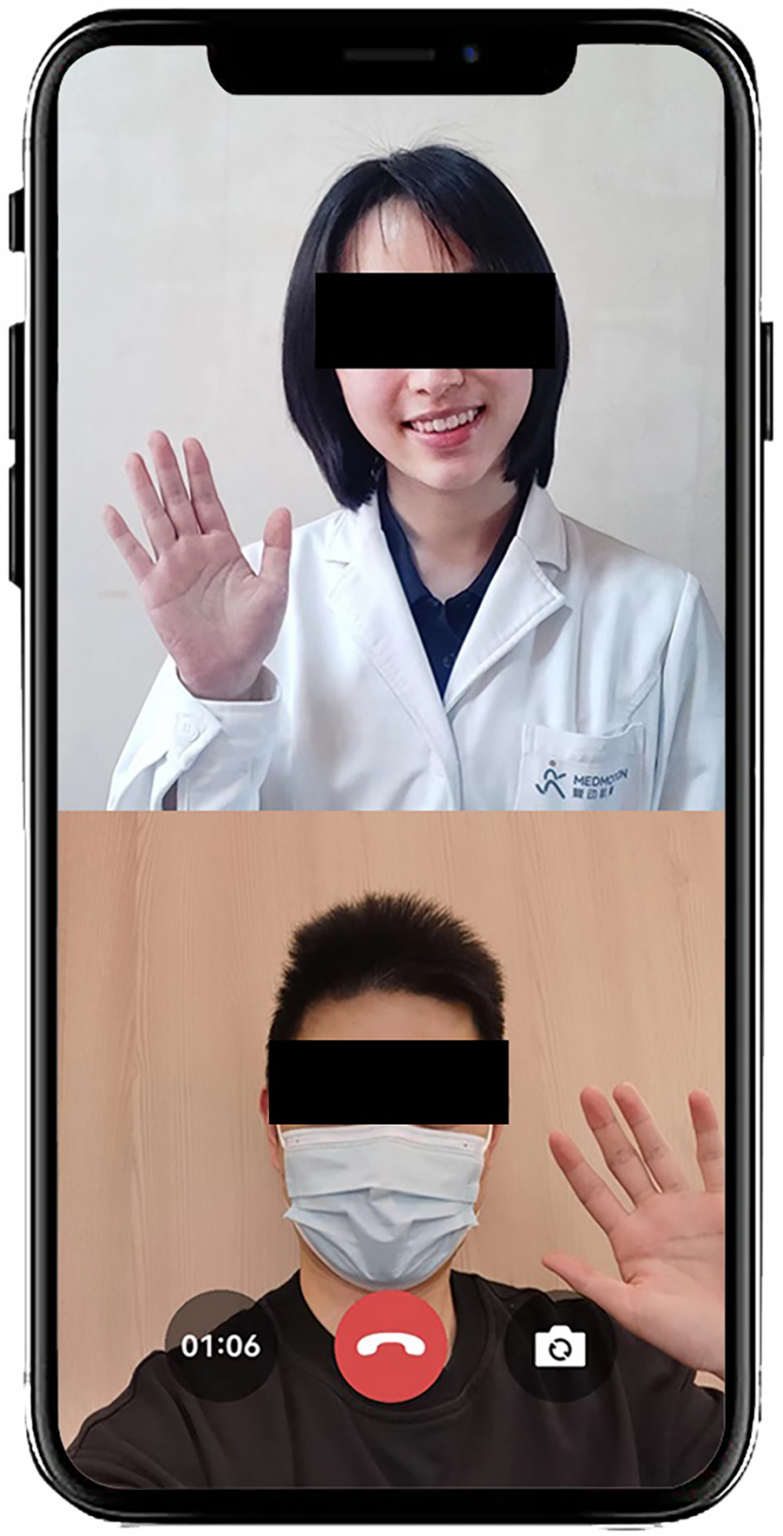
Example of video consultation on the mobile APP.

**Figure 3:**
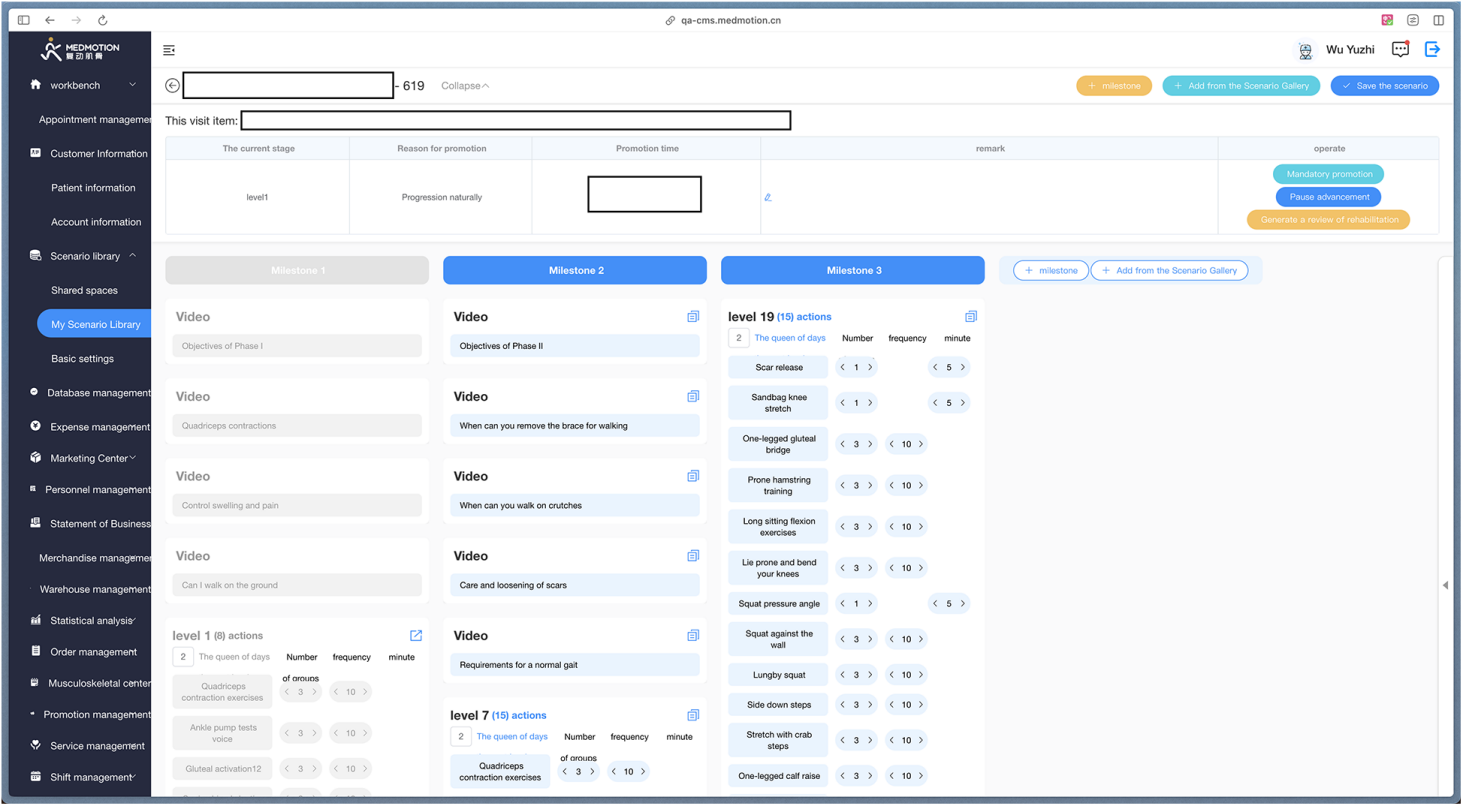
The rehabilitation program prescribed by PT on the backend management system.

The rehabilitation program of the TELE group was delivered through a mobile APP (device: Joymotion R software, Shanghai Medmotion Medical Management Co., Ltd., Shanghai, China.) (11–14), which provided participants with exercise instructions, feedback on their training performance, and real-time two-way video and audio interaction with the PT (Figure 1). The APP was installed by a technician on the same day of the patient's discharge. Internet connection was provided by the patient's home Wi-Fi. PT at the rehabilitation center initiated the conference at the appointed time scheduled with the patient every week (Figure 2). The APP offers daily rehabilitation exercises with detailed instructions and records the exercise completion rates. The rehabilitation program was prescribed by the supervising PT and was assigned to the patient as “daily tasks”. (Figure 3).

Patients in the PT group went to the first rehabilitation hospital in Shanghai after discharge from two trauma centers (Shanghai Jiao Tong University affiliated sixth people's hospital and Shanghai Tongji University affiliated tenth people's hospital) and stayed for one month, patients received a standard rehabilitation program for an allied PT. In the next two months, the patients visited the PT at the outpatient clinic once a week and a 3-session plan were prescribed for one week. The components of the intervention and following home exercises were prescribed according to PT's assessment before and after exercise.

During the intervention, participants received an explanation about the exercises and use of the APP and filled out questionnaires on the day of discharge (E1). The second and third follow-ups were conducted on E2 and E3, including physical tests and filling out questionnaires on the APP or at the clinic.

### Outcome measures

The primary outcome was the QOL subscale of HOOS at 12-weeks follow-up. The HOOS is a self-reported functional questionnaire that evaluated patients' subjective opinions on their hip-related symptoms and functional limitations during a therapeutic process (15). The HOOS has 5 subscales: pain, symptoms, function in activities of daily living (ADL), function in sport and recreation, and hip-related quality of life (QOL). Each question is scored from 0 to 4 on a 5-point Likert scale. A normalized score ranging from 0 to 100 is subsequently calculated for each subscale, with 0 indicating extreme symptoms and 100 indicating no symptoms (15). The content validity of HOOS as well as the test-retest reproducibility has been established in the THA population (15, 16).

The Short Form-12(SF-12) is a multipurpose, short-form general health survey with 12 questions that evaluate functional state and well-being based on the patient's point of view (17, 18). It yields physical component summary (PCS) and mental component summary (MCS) scores.

The timed Up and Go test (TUG) requires the patient to rise from an armchair, walk 3 meters, turn, walk back and sit down in the armchair. It is a valid measurement for evaluating functional ability for patients who has problems with lower extremity (19).

The original 14-item Berg Balance Scale (BBS) has been reduced to a 7-item BBS, with the purpose of gathering similar information while reducing the time and complexity of administration (20). The short BBS consists of seven of the original BBS tasks, scored with the original scale. Compared to the old version of BBS, the short version of BBS took great less time to administer, with good validity and reliability (20).

### Sample size

The sample size was calculated based on the data of primary outcomes (subscales of the Hip disability and Osteoarthritis Outcome Score, HOOS) (16), employing the noninferiority power calculation described by Jones et al. (21).

The subscales of pain, symptoms, and quality of life (QOL) were calculated separately for the sample size. The minimal clinically important improvement (MCII) values determined by Paulsen et al. (22) were used for noninferiority margin (QOL 17, pain 24, symptoms 23). The intervention will be accepted as equivalent if the difference between the two groups is less than MCII. The common standard deviation (SD) was 18 (0–100 scale), according to previous studies (23). Calculations were based on 90% power and a type-I error of 2.5% (*α* = 0.025, one-tailed). The values of QOL yielded the largest sample size of 25 per group, thus we set a sample size of 31 per group based on a 20% of dropout rate.

### Randomization

A computer-generated randomization list (SAS Proc Plan, SAS/STAT 9.3; SAS Institute, Cary, North Carolina) was prepared by the statistician and given to the study's clinical coordinator of each site in a series of sealed envelopes. Afterward, the study coordinator proceeded to randomization in the patient's presence.

### Blinding

All evaluators and investigators were blinded to group assignment for the entire duration of the study. Decisions related to data analyses were taken while investigators were still unaware of the group assignment. However, blinding subjects and clinicians was not possible, considering the nature of the intervention.

### Statistical analysis

The groups were first compared on baseline characteristics. Differences in continuous variables between groups were assessed using independent-sample *t*-tests or Mann-Whitney *U* tests, depending on the results of the Shapiro-Wilk test for normality. For categorical variables, the FREQ (frequency and contingency) procedure was used to perform chi-square tests, or Fisher's exact tests when chi-square testing was invalid. The main research hypothesis posited that the mean QOL score gain from baseline (E1) to the last follow-up (E3) in the intervention group would not be inferior to that in the control group. In the primary analysis, only subjects who participated in all evaluations and attended at least 75% of the intervention sessions were included in the per-protocol analysis. To control for the risk of Type I errors due to multiple comparisons, *p*-values were adjusted using the false discovery rate (FDR) method. The adverse events intention-to-treat population was also assessed.

For normally distributed data, means and standard deviations (SD) are presented, with comparisons made using *t*-tests. Effect sizes for primary outcomes were calculated using Cohen's d. For non-normally distributed data, medians and interquartile ranges (IQR) are reported, with comparisons conducted using Mann–Whitney *U* tests. Effect sizes for primary outcomes in this case were determined using Cliff's Delta. All statistical analyses were performed using SAS 9.3, with a significance level set at 0.05 (two-sided).

## Results

### Demographic characteristics

**Figure 4:**
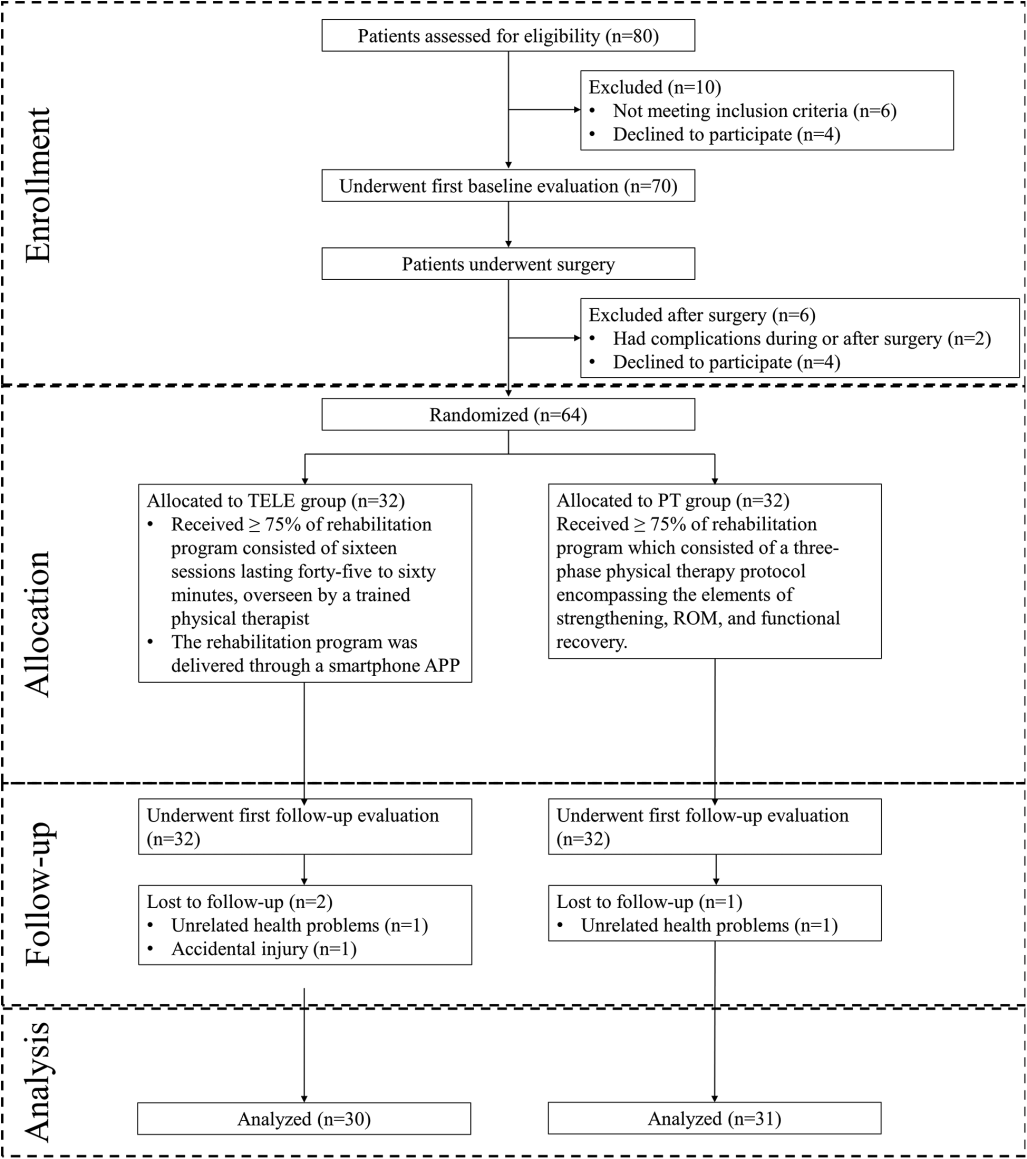
Trial profile of PT vs. TELE group of participants.

Eighty patients were screened before surgery, and sixty-four were randomized after surgery. Three participants (2 in the TELE group, and 1 in PT group) failed to complete the study due to accidental injury (*n* = 1) and unrelated reasons (*n* = 2). The remaining 61 participants completed the study and the entire follow-up survey. The reasons for exclusion and drop-out are specified in Figure 4.

An overview of the demographic characteristics of the two groups is presented in Table 1. No significant differences between groups were found.

**Table 1:** Demographics and baseline characteristics of the patients.

Demographic	PT group (*n* = 31)	TELE group (*n* = 30)	*P*-value
Age (yr)	70 [63, 73]	69 [66, 72]	0.89
Gender (%)			0.35
Female	15 (48.4)	10 (33.3)	
Male	16 (51.6)	20 (66.7)	
BMI (kg/m^2^)	23.6 [22.5, 27.5]	24.0 [22.1, 26.2]	0.81
Smoking (%)			0.35
Current smoker	6 (19.4)	5 (16.7)	
Never smoke	16 (51.6)	11 (36.7)	
Used to smoke	9 (29.0)	14 (46.7)	
Diabetes (%)			0.37
BG < 11.1 mmol/L	19 (61.3)	14 (46.7)	
BG ≥ 11.1 mmol/L	12 (38.7)	16 (53.3)	
Side of Hip (%)			0.90
Left	16 (51.6)	14 (46.7)	
Right	15 (48.4)	16 (53.3)	
Living alone (%)			0.92
No	19 (61.3)	17 (56.7)	
Yes	12 (38.7)	13 (43.3)	

Data are presented as median [IQR] and were analyzed using Mann–Whitney *U* tests. Categorical variables are presented as counts with percentages in parentheses and were analyzed using chi-square tests.

BMI, body mass index; BG, blood glucose.

### Physical function

Significant improvements in the results of the TUG and BBS were noted from the follow-up time point E2 to E3, in both TELE and PT groups. The results of functional measurements are presented in Table 2 and the gain from E2 to the last follow-up (E3) was shown in Table 3. Patients were not tested for TUG and BBS at the time point of discharge. No significant differences were found in the TUG at E2 and E3 between the two groups. The BBS also showed no significant differences at each time point between the two groups. The gains from E2 to E3 showed no significant differences (Table 3).

**Table 2:** Physical function and general health outcome at baseline, week 4 and 12.

Outcome	PT group (*n* = 31)	TELE group (*n* = 30)	*P*-value	Effect size
HOOS QOL^a^				
Baseline	12.5 [6.3, 12.5]	12.5 [6.3, 12.5]	0.83	0.029
Week 4	49.0 (5.6)	48.4 (5.9)	0.66	0.114
Week 12	77.0 (5.0)	76.9 (5.3)	0.92	0.026
HOOS Pain				
Baseline	17.5 [11.2, 20.0]	15.0 [7.5, 20.0]	0.92	
Week 4	67.5 [65.0, 70.0]	67.5 [65.0, 70.0]	0.92	
Week 12	85.0 [78.8, 90.0]	85.0 [80.0, 90.0]	0.79	
HOOS Symptoms				
Baseline	30.0 [25.0, 30.0]	30.0 [25.0, 30.0]	0.79	
Week 4	59.8 (7.8)	58.3 (8.4)	0.49	
Week 12	80.0 [75.0, 85.0]	80.0 [80.0, 85.0]	0.93	
HOOS ADL				
Baseline	5.0 (2.6)	5.6 (2.7)	0.33	
Week 4	60.6 [52.4, 65.8]	60.9 [52.7, 65.9]	0.89	
Week 12	79.7 (8.3)	80.6 (8.3)	0.69	
HOOS Sport/Rec				
Baseline	0	0	N/A	
Week 4	7.9 (4.5)	7.9 (4.0)	0.96	
Week 12	53.3 (4.5)	52.9 (4.3)	0.78	
SF-12 PCS				
Baseline	21.3 [19.6, 22.6]	21.1 [19.5, 22.1]	0.71	
Week 4	39.4 [37.3, 41.0]	38.8 [36.9, 40.0]	0.29	
Week 12	50.4 [47.5, 52.0]	49.8 [47.8, 50.9]	0.43	
SF-12 MCS				
Baseline	21.3 [18.6, 22.4]	20.0 [17.9, 22.4]	0.55	
Week 4	41.0 [37.5, 44.4]	39.8 [36.8, 43.6]	0.15	
Week 12	49.9 [47.6, 53.1]	50.1 [47.6, 53.3]	0.78	
TUG (s)				
Baseline	N/A	N/A	N/A	
Week 4	26.2 [23.5, 28.2]	27.8 [25.5, 29.1]	0.14	
Week 12	18.2 [17.1, 19.9]	18.6 [15.6, 19.8]	0.62	
Berg Balance Scale				
Baseline	N/A	N/A	N/A	
Week 4	19.5 [18, 22]	19.3 [17, 21]	0.85	
Week 12	38.3 [35, 42]	38.3 [35, 410]	0.96	

N/A, Not applicable.

Normally distributed data are presented as mean values with standard deviations (SD) in parentheses, analyzed using *t*-tests, with effect sizes calculated using Cohen's d for primary outcomes. Non-normally distributed data are presented as medians with interquartile ranges (IQR) and analyzed using Mann–Whitney *U* tests, with effect sizes determined by Cliff's Delta for primary outcomes.

HOOS, Hip Disability and Osteoarthritis Outcome Score, scale of 0–100 (0 = extreme symptoms, 100 = no symptoms); ADL, activities in daily living; Sport/Rec, function in sport and recreational activities; QOL, Hip-related quality of life; SF-12, 12-item Short Form Health Survey, scale of 0–100 (higher score = better perceived health or functioning); PCS, physical component summary; MCS, mental component summary; TUG, timed up and go test (measured by seconds).

^a^ Primary outcome measure.

**Table 3:** Change of physical function and general health outcome from baseline to follow-up.

Outcome	PT group (*n* = 31)	TELE group (*n* = 30)	*P*-value	Effect size
HOOS QOL^a^				
From baseline to week 4	37.5 [34.4, 43.8]	37.5 [37.5, 43.8]	0.87	0.025
From week 4 to week 12	25.0 [25.0, 31.3]	25.1 [25.0, 31.3]	0.69	−0.058
From baseline to week 12	68.7 [62.5, 73.1]	68.7 [62.5, 69.0]	0.70	0.058
HOOS Pain				
From baseline to week 4	50.0 [50.0, 53.8]	52.5 [50.0, 55.0]	0.60	
From week 4 to week 12	17.5 [13.8, 20.0]	17.5 [15.0, 20.0]	0.60	
From baseline to week 12	67.5 [67.5, 70.0]	70.0 [67.5, 70.0]	0.09	
HOOS Symptoms				
From baseline to week 4	31.9 (3.8)	30.7 (4.8)	0.29	
From week 4 to week 12	22.5 [16.2, 26.2]	25.0 [15.0, 27.5]	0.36	
From baseline to week 12	55.0 [50.0, 55.0]	55.0 [50.0, 55.0]	0.80	
HOOS ADL				
From baseline to week 4	54.8 (8.4)	54.4 (7.6)	0.86	
From week 4 to week 12	20.0 (11.1)	20.5 (10.6)	0.84	
From baseline to week 12	74.8 (8.9)	74.9 (8.4)	0.94	
HOOS Sport/Rec				
From baseline to week 4	7.9 (4.5)	7.9 (4.0)	0.96	
From week 4 to week 12	45.4 (7.4)	45.0 (5.8)	0.83	
From baseline to week 12	53.3 (4.5)	52.9 (4.3)	0.78	
SF-12 PCS				
From baseline to week 4	18.0 (3.6)	17.6 (3.0)	0.64	
From week 4 to week 12	10.9 (4.0)	11.1 (3.1)	0.81	
From baseline to week 12	28.8 (3.2)	28.7 (3.1)	0.82	
SF-12 MCS				
From baseline to week 4	20.5 (4.2)	19.8 (4.7)	0.54	
From week 4 to week 12	9.0 (5.0)	10.2 (4.9)	0.36	
From baseline to week 12	29.0 [27.2, 33.1]	30.4 [28.0, 32.8]	0.59	
TUG (s)				
From baseline to week 4	N/A	N/A	N/A	
From week 4 to week 12	−7.8 (3.7)	−9.2 (3.3)	0.11	
From baseline to week 12	N/A	N/A	N/A	
Berg Balance Scale				
From baseline to week 4	N/A	N/A	N/A	
From week 4 to week 12	18.9 (4.6)	19.1 (5.2)	0.9	
From baseline to week 12	N/A	N/A	N/A	

Data are mean change scores from baseline to follow-up.

N/A, Not applicable.

Normally distributed data are presented as mean values with standard deviations (SD) in parentheses, analyzed using *t*-tests. Non-normally distributed data are presented as medians with interquartile ranges (IQR) and analyzed using Mann–Whitney *U* tests, with effect sizes determined by Cliff's Delta for primary outcomes.

HOOS, Hip Disability and Osteoarthritis Outcome Score, scale of 0–100 (0 = extreme symptoms, 100 = no symptoms); ADL, activities in daily living; Sport/Rec, function in sport and recreational activities; QOL, Hip-related quality of life; SF-12, 12-item Short Form Health Survey, scale of 0–100 (higher score = better perceived health or functioning); PCS, physical component summary; MCS, mental component summary; TUG, timed up and go test (measured by seconds).

^a^ Primary outcome measure.

### Self-reported measurements

The results of self-reported measurements are shown in Table 2 and the score gains are shown in Table 3. The results of the TELE group and the PT group were close. Each subscale of the HOOS has similar results between the two groups. Also, there were no differences at any time point of the SF-12 between groups, in terms of the physical and mental components. The differences between the groups concerning the HOOS and SF-12 gains from baseline values were of no statistical significance at any time point (E2-E1, E3-E1, E3-E2) in each subscale. The *p*-values were adjusted using the FDR method. The adjusted *p*-values for Table 2 are 0.96, while those for Table 3 are 0.98. Effect sizes for primary outcomes were small, indicating a slight tendency for the PT group to have higher values than the TELE group, though this is unlikely to have practical significance.

### Adverse events

During the follow-up period, a similar proportion of participants in both groups reported adverse events. No serious events were related to the telerehabilitation intervention, while one minor event was possibly related to the standard intervention (Table 4). The proportions of participants lost to follow-up were equivalent in both groups, with most losses occurring at the final follow-up survey (Figure 4).

**Table 4:** Adverse events and serious adverse events.

Adverse events	PT group (*n* = 32)	TELE group (*n* = 32)
Patients with adverse events [no. (%)]	3 (9.4)	4 (12.5)
Events related to study therapy (no.)	0	1^a^
Events unrelated to study therapy (no.)	4	4
Type of event (no.)		
Involved knee		
Pain	3	2
Bruising	0	0
Swelling	0	1
Signs of infection (swelling, redness, heat, or pus)	0	0
Problems with wound-healing	0	0
Mobilization under anesthesia	0	0
Other		
Fall with minor symptoms	1	0
Nausea and dizziness	0	0
Back pain	0	1
Anxiety about knee recovery	0	0
Serious adverse events		
Patients with serious adverse events [no. (%)]	1 (3.1)	2 (6.3)
Events related to study therapy (no.)	1	2
Events unrelated to study therapy (no.)	0	0
Type of event (no.)		
Death	0	0
Degradation of the general condition	0	0
Hip fracture due to fall	0	1^b^
Gastrointestinal disorder	1^b^	0
Cardiac arrhythmia	0	1^b^
Spinal surgery	0	0

^a^One patient fell during intervention with minor consequent symptoms.

^b^Events related to hospitalization.

## Discussion

The purpose of this study was to evaluate the effectiveness of a home-based rehabilitation program delivered using a mobile APP. To that end, the effectiveness of this program was compared with traditional face-to-face PT care in China. We hypothesized that a home-based rehabilitation program could offer a non-inferior effective alternative to traditional face-to-face PT care for patients after THA.

### Physical function

Significant improvements were noted considering the objective outcomes, the telerehabilitation program seemed to have had considerable effects on the TUG test and the Berg Balance test at the end of the 12-week program. The self-assessment outcomes further support these improvements, thus verifying our hypothesis.

Different from patients who underwent open reduction and internal fixation after traumatic fracture, patients with THA are more suitable for telerehabilitation because of the unified surgical methods and the standard and unified postoperative rehabilitation. In addition, many clinical studies with large sample size have been carried out in western countries for patients with THA or TKA (total knee arthroplasty) in recent decades (5–7, 24, 25), with similar results supporting home-based telerehabilitation to be a reliable alternative to traditional PT-based postoperative care for patients after THA.

### Implications for postoperative THA rehabilitation

In 2014, North American Expert Consensus Group on best practices for acute rehabilitation after total hip replacement recommended that supervised rehabilitation interventions be provided by trained health professionals (physical therapists) shortly after discharge from an acute care facility (26). More than 75 percent of panelists also recommended individual therapy as opposed to group therapy in an outpatient setting or at home, while acknowledging major differences in rehabilitation practices and program delivery patterns worldwide (26). In Canada, Australia, and the United States, at least one-third of the patients receive some rehabilitation through face-to-face home-care services (26–30). But China is still short of trained physical therapists, approximately only 5% of postoperative THA or TKA patients receive face-to-face in-hospital rehabilitation services (31), not to mention face-to-face home-care services. Telerehabilitation, as a new method of service delivery, has promised to improve the situation caused by the shortage of physical therapists in China.

Several factors may have contributed to the recovery of the telerehabilitation group. The nature of telerehabilitation interventions, which rely more on educating patients about self-applied mobilization techniques and place greater emphasis on exercise, may provide participants with more opportunities to the self-treat outside of formal physiotherapy sessions. The higher dependence on education in the telerehabilitation group may have contributed to the higher technical proficiency in the home exercise program (32). More education has been shown to benefit so-called internal control points, which are considered an important factor in patient compliance (33, 34). As hip osteoarthritis and hip fractures occur more frequently in older adults, most participants in this study required the assistance of their children to complete the operation of the devices. With the popularity of electronic equipment, we believed that the acceptance of telerehabilitation will increase in the next generations.

The successful implementation of telerehabilitation for THA patients is influenced by several socio-economic and infrastructural factors that can pose significant challenges, particularly in rural or underserved communities. Patients in these areas often have lower levels of education and limited internet access, which can hinder their ability to effectively engage with digital health technologies. Moreover, the lack of familiarity with mobile devices and applications may reduce their confidence in using telerehabilitation services, thereby impacting compliance and outcomes (35). These limitations necessitate the development of tailored educational programs that can enhance digital literacy among THA patients (36). Additionally, improving internet infrastructure or providing alternative means of communication are critical steps towards making telerehabilitation a feasible option for all patients, regardless of their geographic or socio-economic status.

### Study strengths and limitations

To date, we are aware of no randomized controlled trials on the effectiveness of in-home telerehabilitation after total hip arthroplasty that have been published in China, and only three randomized controlled trials on the effectiveness of in-home telerehabilitation after total knee and total hip arthroplasty have been published in Canada and Australia (6, 7, 25). The data in this study provide an essential reference for a more comprehensive and in-depth study on the effectiveness of telerehabilitation in different countries and regions.

Several limitations in the current study are acknowledged. Because the long-term effects of this rehabilitation program are unknown, the limited follow-up period of 12 weeks has implications for the interpretation of the results. Therefore, future studies must take advantage of the extended follow-up period to better characterize the long-term effects of this alternative service delivery model. Finally, the quality of the Internet connection between telerehabilitation units is easily monitored in this controlled environment, a factor that can be variable when delivered in a patient's home. For these reasons, this study should be viewed as a proof-of-principle study, and future studies should be conducted in communities and homes where patients are isolated to explore the impact of these factors.

## Conclusions

Our study proved that mobile-based in-home telerehabilitation programs are non-inferior to in-hospital face-to-face rehabilitation for elderly patients who underwent THA due to femoral neck fracture. In addition, adherence to the telerehabilitation program showed that the novel technology was accepted well and could be an alternative to the conventional in-person rehabilitation program.

## Data Availability

The raw data supporting the conclusions of this article will be made available by the authors, without undue reservation.
